# Patients with COVID-19 Pneumonia Admitted to an Intensive Care Unit (ICU) at a Community Hospital in Flint, Michigan, in Early 2020: Characteristics and Mortality

**DOI:** 10.51894/001c.89371

**Published:** 2023-12-05

**Authors:** Atefeh Kalantary,, Olga J. Santiago-Rivera, Arunima Dutta, Chace Davies, Bilal Malik, Parul Sud, Ibrahim Al-Sanouri

**Affiliations:** 1 Medical Director Menalam Health Services PC, Simi Valley, CA; 2 Graduate Medical Education Internal Medicine Residency Program McLaren Health Care; 3 Internal Medicine Virginia Mason Franciscan Health, Seattle, WA; 4 College of Osteopathic Medicine Michigan State University; 5 Internal Medicine Residency Program McLaren Flint Hospital; 6 Internal Medicine Residency Program Director McLaren Flint Hospital; 7 Michigan State University; 8 Pulmonary Critical Care Fellowship McLaren Flint Hospital

**Keywords:** KEYWORDS: COVID-19, Flint, pneumonia, in-hospital mortality, Intensive Care Unit

## Abstract

**INTRODUCTION:**

Despite the many studies conducted on the factors affecting mortality in patients with COVID-19, there is ongoing debate about the role of race as a risk factor. Several studies have reported a geographic and racial disparity in COVID-19 cases in Michigan. This study aimed to examine the characteristics of the 2020 first cohort of Intensive Care Unit (ICU) COVID-19 patients admitted to a community teaching hospital in Flint, Michigan, and to determine the factors associated with ICU mortality, including race.

**METHODS:**

This cross-sectional study included adult patients (≥ 18 years) with severe COVID-19 pneumonia admitted to the ICU between March and May 2020. Potential risk factors associated with ICU mortality included demographic characteristics, comorbidities, treatments, and complications.

**RESULTS:**

The study sample consisted of N = 48 patients, aged 24–85 years, (mean 59.7; SD = 12.8); 56.2% (n=27) were male and 51.1% (n=24) were Black adults. The mortality rate was 51.1%. Age (aOR 1.1, 95% CI [1.01, 1.20]; p =0.03), type 2 diabetes (aOR 5.7, 95% CI [1.2, 29.1]; p =0.03), and essential hypertension (aOR 6.2, 95% CI [1.1, 34.5]; p =0.04) were all found to have statistically significant independent associations with increased risk of ICU mortality in this study sample. On the other hand, race was not found to be associated with ICU mortality.

**CONCLUSIONS:**

These findings support the literature regarding the association of comorbid conditions, including type 2 diabetes and hypertension, with poorer outcomes in ICU hospitalized patients with severe COVID-19 pneumonia. This study provides insight into mortality of an ICU patient cohort earlier on during the COVID-19 pandemic in Flint, Michigan.

The Center for Disease Control (CDC) confirmed the first case of COVID-19 in the United States (US) on January 20, 2020.^1^ Four months later, at the end of May, US COVID-19 deaths surpassed 100,000.^1^ At the end of 2020, 385,676 deaths were attributed to COVID-19 in the US.^2^ The first wave of COVID-19 in the US has been defined in similar time frames in the literature, mostly between March and May 2020.^3–6^ Previous studies have attempted to identify the factors that contributed to an increase in the first wave COVID-19 mortality. Hypertension, diabetes, age, and cardiovascular disease have been consistently associated with a greater risk of mortality among hospitalized COVID-19 patients.^4–7^ These same characteristics were determined to be risk factors for intensive care unit (ICU) admission during the first COVID-19 wave (March 1 to May 2, 2020).^5^

It has been estimated that 29% of adult patients received ICU-level care in academic hospitals and their affiliates in the United States during the initial 6-month period of the pandemic (March to August 2020).^7^ Similarly, a study of the COVID-19-Associated Hospitalization Surveillance Network found that 32% of patients needed ICU admission between March 1 to May 2, 2020.^5^ Multiple comorbidities and underlying risk factors, including asthma, age, body mass index (BMI), hypertension, chronic renal failure, and diabetes have all been identified as factors associated with COVID-19 ICU mortality.^8–11^

The literature on ICU mortality, during the first waves of COVID-19, was focused on patients from other countries,^10,11^ or on academic health care networks in the United States.^8^ These studies’ findings may not be generalizable to other ICU settings, such as those in community hospitals. In addition, the impact demographic context (e.g., residing in underserved neighborhoods with financial insecurity, race, etc.) has been associated with incidence of COVID-19^12^ and should be included when examining ICU mortality risk factors.

## COVID-19 in Michigan

In Michigan, 5,087 persons died during the first wave of COVID-19 (March to May 2020).^3^ A weekly average of 4,000 hospitalizations occurred in April 2020, the month with the highest first-wave death and hospitalizations.^3^ The initial COVID-19 wave disproportionately affected the Black population.^13^

Several studies have reported a geographic and racial disparity in COVID-19 cases in Michigan.^14–18^ For example, Genesee County, Flint and the surrounding areas, where Blacks/African Americans [AA] make up 54% of the population,^19^ had the highest incidence of COVID-19 during the first wave.^14^ Flint has been described as having a vulnerable population since 37.3% of its residents live below the poverty line, compared to the US national average of approximately 11%.^19^ From the beginning of the pandemic until June 2020, Black/AA residents in Genesee County were 3.3 times more likely than White residents and other races to have COVID-19.^14^ Similarly, a study conducted in Oakland County, Michigan, reported that zip codes with a higher concentration of Black/AA residents were disproportionately affected by COVID-19, and that between April 3 to May 16, 2020, Black/AA residents experienced a greater increase in COVID-19 incidence rates than other races.^15^ Demographic characteristics of geographic areas disproportionately affected by new/emerging infectious diseases provide a way of identifying vulnerable populations. It also provides a way to be prepared for future outbreaks/epidemics and where to allocate public health efforts during the early stages of the disease.

Even though there has been a consistent association demonstrated between race (i.e., Black/AA residents) and the incidence of COVID-19 in Michigan, there are conflicting studies’ findings regarding the relationship between race and COVID-19 case mortality rate and fatality rate.^15,16,18^ Such differences can be partially attributed to the studies’ target populations (percentage of Blacks/AA in a zip code vs. percentage of Blacks/AA in hospital admissions); the mortality measurements of the study outcomes (fatality rate vs. mortality rate); and the time frames of case analysis (e.g., COVID-19 waves). For instance, Akambi and colleagues analyzed data from Oakland County between April 3, 2020 and May 16, 2020, reporting no association between the proportion of Black/AA residents in a zip code with case fatality.^15^ Similarly, Suleyman and colleagues did not find an association between race and *mortality rate*; their study period was from March 9 to March 27, 2020, and their study population consisted of 463 consecutive COVID-19 patients evaluated at Henry Ford Health System in the metropolitan Detroit, Michigan area.^16^ In contrast to Akambi’s and Suleyman’s studies,^15,16^ a study conducted in Michigan using death certificates and surveillance data from March 16 to October 26, 2020, found that Black/AA individuals had a higher risk of COVID-19 mortality than their White counterparts.^18^ No studies were found that describe COVID-19 pneumonia ICU admissions or the factors associated with ICU mortality, including the racial differences, among COVID-19 pneumonia patients in Flint, Michigan.

### Purpose of Study

This study aimed to describe the sociodemographic characteristics, comorbidities, treatments received, and complications of patients diagnosed with severe COVID-19 pneumonia and required admission to the ICU for high-acuity management during the first wave of COVID-19 cases in a community hospital in Flint, Genesee County, Michigan. In addition, we aimed to identify patient-level risk factors associated with ICU in-hospital mortality. Considering that the first wave of COVID-19 has been characterized by a high percentage of patients requiring ICU level of care, with Black/AA populations being disproportionately affected, and the geographic gaps of COVID-19 cases in Michigan, the investigators hypothesized that there will be differences in ICU in-hospital mortality by race in this study population.

## METHODS

### Study setting and study sample

The setting of this cross-sectional study was a community teaching hospital in Flint, Michigan, with 376-operational beds, and an average of 17,000 general admissions and 3,240 ICU admissions per year. The racial distribution among admitted patients is around 76% White and 21% Black/AA. Similarly, the overall distribution of patients admitted to ICU is around 78% White and 18% Black/AA.

The study sample consisted of adult inpatients (≥ 18 years old) diagnosed with severe COVID-19 pneumonia and admitted to the ICU during the first wave of COVID-19 (from March 1 to May 31, 2020). The first wave in this study is defined by the months with the first patient and the highest proportion of COVID-19 ICU admissions. Severe COVID-19 pneumonia was defined per World Health Organization (WHO) as infection requiring supplemental oxygen or ventilatory support and/or oxygen saturation less than 94% on room air in conjunction with dyspnea. Patients without COVID-19 were excluded. The medical records department provided a list of patients admitted during the study period. All data was collected through a retrospective electronic medical record chart review. A total of 200 ICU admissions were reviewed, and 152 patients were excluded for not meeting the inclusion criteria. The final study sample consisted of 48 severe COVID-19 pneumonia ICU patients.

### Study measures

The primary study outcome was ICU mortality in patients with severe COVID-19 pneumonia. The potential risk factors for in-hospital mortality among ICU COVID-19 patients included sociodemographic characteristics such as age, sex, race, health behaviors (i.e., current smoking), comorbidities, health status indicators during hospitalization, treatments, and complications. The demographics characteristics were self-reported, and comorbidities were identified either by past medical history or current clinical interview/assessment.

Comorbidities included for example, hypertension, diabetes, coronary artery disease, and obesity. Furthermore, the Charlson Comorbidity Index (CCI), a weighted index that has been shown to predict the risk of death within one year of hospitalization with specific comorbid conditions, was also calculated.^20^ In this study, the CCI ranged from a score of zero (no comorbidities) to 11; the higher the score, the more likely it is that the predicted outcome will be mortality.

In terms of treatments and complications, end organ damage including acute kidney injury (AKI), myocardial infarction (MI), pulmonary embolism (PE) among others were included. The study protocol was reviewed and approved by the McLaren Health Care Institutional Review Board (protocol number 2020-00031).

### Statistical Analyses Plan

The statistical approach used means, medians, frequencies, and percentages to estimate the characteristics of the study sample. The analyses included only observations without missing data. To investigate potential associations between in-hospital ICU mortality and patient characteristics, we used the Fisher’s exact test with categorical variables of interest (e.g., sex and race) and the Wilcoxon rank-sum test for continuous variables (e.g., age). The statistical method used in the subsequent analysis involved using unadjusted and adjusted logistic regression.

The final model consisted of a multivariate logistic regression analysis, with in-hospital mortality serving as the primary outcome. An investigator-guided stepwise procedure was performed to determine the most parsimonious model. The initial regression model included sociodemographic characteristics that have been associated with in-hospital mortality and COVID (i.e., age, race, sex, and comorbidity index), along with variables that had a *P*-value of ≤ 0.20 in the bivariate analyses. Additional variables were incorporated into the model while testing for statistical significance and assessing collinearity. In this analysis, the accuracy of the study estimates was examined with an emphasis on 95% confidence intervals, and *P*-values were provided to aid with interpretation. All analyses were performed (OJS) using Stata 15.1 SE.^21^

## RESULTS

### Description of the study sample

Our study sample consisted of N = 48 ICU patients with severe COVID-19 pneumonia, ranging in age from 24 to 85, with a mean age of 59.7 years (SD = 12.8 years); 55% (n = 27) were men and 51.1% (n = 24) were Black/AA patients. Most of the patients (n = 30, 66.7%) were nonsmokers, while 83.3% (n = 40) were overweight or obese (Table 1).

The mean CCI was 3.37 (SD 3.0, range 0 to 11). The two most prevalent co-existing conditions were essential hypertension (70.2%; 33/47); and diabetes (Type 1 or Type 2) (45.8%; 22/48). Type 2 Diabetes was present in 41.7% (20/48) of the study sample. In terms of hematological abnormalities, the most prevalent were anemia (88.0%; 40/46), thrombocytopenia (46.7%; 21/45), and bi-thrombocytopenia-pancytopenia (41.3%; 19/46). The average length of stay for patients in the ICU was 18.2days (SD = 16.7 days), ranging from 0 to 62 days. Thirty-eight (82.6%; 38/46) patients required invasive ventilation. Acute respiratory distress syndrome (ARDS) was present in 87% (38/44) of the study sample, with moderate ARDS accounting for 63.2% (24/28) and severe ARDS for 21% (8/38) of cases. Some hospital-related complications included elevated liver function tests (84.8%; 39/46) and acute kidney injury (AKI) (76.6%; 36/47). One case of ESRD and one case of liver disease were not included in the bivariate analyses.

### Primary Outcome

The primary study outcome was ICU mortality in patients with severe COVID-19 pneumonia. In this study sample, there was a 52% (25/48) mortality rate. Table 1 and Table 2 present the association between ICU mortality and other covariates of interest. For example, bivariate association analyses demonstrated that patients with essential hypertension, type 2 diabetes, and high CCI index had significantly higher ICU mortality (Table 1) than their counterparts. Similarly, the results of the unadjusted logistic regressions suggested that essential hypertension and type 2 diabetes were associated with a higher risk of ICU mortality (Table 2).

The initial adjusted logistic regression model tested for statistical significance and evaluated for interactions and collinearity included age, race, sex, and comorbidity index. While the final adjusted logistic regression model included age, sex, essential hypertension, and diabetes type 2 (Table 2). The factors independently associated with in-hospital mortality among severe COVID-19 pneumonia patients who required admission to the ICU from March 1st to May 31st, 2020, were essential hypertension (adjusted odd ratio [aOR] 6.2; *P* = 0.04), type 2 diabetes (aOR 5.7; *P* = 0.03), and age (aOR 1.1; *P* = 0.03).

**Table 1.** Characteristic of the study sample (N=48). Bivariate association between study characteristics and ICU mortality.

Patient Characteristic	Obs.	Total N=48 n (%)	Deaths n=25 n (%)	Survival n=23 n (%)	p-value
Age Mean; years (SD); Range 24-84	47	59.7 (12.8)	62.9 (11.1)	56.1 (13.8)	0.11^a^
Sex Male Female	48	27(56.3) 21 (43.7)	15 (60.0) 10 (40.0)	12 (52.2) 11 (47.8)	0.77^b^
Race White Black/African American	47	23 (48.9) 24 (51.1)	14 (58.3) 10 (41.7)	9 (39.1) 14 (60.9)	0.25
Location before admission Home Nursing Home	46	38 (82.6) 8 (17.4)	18 (75.0) 6 (25.0)	20 (90.9) 2 (9.1)	0.25
Smoking status Never smoked Current/former smoker	45	30 (66.7) 15 (33.3)	16 (69.6) 7 (30.4)	14 (63.6) 8 (36.4)	0.76
*Comorbidities*					
Overweight or obese (BMI 25 kg/m^2^) No Yes	48	8 (16.7) 40 (83.3)	3 (12.0) 22 (88.0)	5 (21.7) 18 (78.3)	0.45
Charlson Comorbidity Index (CCI), mean (SD) (n=46), range 0-11	46	3.37 (3.0)	4.09 (2.7)	2.65 (3.4)	**0.03**
Diabetes No Yes	48	26 (54.2) 22 (48.8)	9 (36.0) 16 (64.0)	17 (73.9) 6 (26.1)	**0.01**
Diabetes Type 2 No Yes	48	28 (58.3) 20 (41.7)	10 (40.0) 15 (60.0)	18 (78.3) 5 (21.7)	**0.01**
Anemia No Yes	46	6 (13.0) 40 (87.0)	3 (12.5) 21 (87.5)	3 (13.6) 19 (86.4)	>0.99
Essential Hypertension No Yes	47	14 (29.8) 33 (70.2)	4 (16.0) 21 (84.0)	10 (45.4) 12 (54.6)	**0.05**
Chronic kidney disease (CKD) No Yes	48	37 (77.1) 11 (22.9)	19 (76.0) 6 (24.0)	18 (78.3) 5 (21.7)	>0.99
Cardiovascular disease No Yes	48	41 (85.4) 7 (14.6)	20 (80.0) 5 (20.0)	21 (91.3) 2 (8.7)	0.42
Chronic obstructive lung disease No Yes	48	43 (89.6) 5 (10.4)	22 (88.0) 3 (12.0)	21 (91.3) 2 (8.7)	>0.99
Immunocompromised No Yes	48	44 (91.7) 4 (8.3)	23 (92.0) 2 (8.0)	21 (91.3) 2 (8.7)	>0.99
Cancer No Yes	48	45 (93.7) 3 (6.3)	25 (100) -	20 (87.0) 3 (13.0)	0.10
*Complications*					
Elevated liver function No Yes	46	7 (15.2) 39 (84.8)	4 (17.4) 19 (82.6)	3 (13.0) 20 (87.0)	>0.99
Continue Renal Replacement Therapy No Yes	47	32 (68.1) 15 (31.9)	15 (60.0) 10 (40.0)	17 (77.3) 5 (22.7)	0.23
Acute kidney injury No Yes	47	11(23.4) 36 (76.6)	3 (12.5) 21(87.5)	8 (34.8) 15 (65.2)	0.09
Bi-thrombocytopenia/pancytopenia No Yes	46	27 (58.7) 19 (41.3)	13 (54.2) 11 (45.8)	14 (63.6) 8 (36.4)	0.56
Thrombocytopenia No Yes	45	24 (53.3) 21 (46.7)	10 (43.5) 13 (56.5)	14 (63.6) 8 (36.4)	0.24
Non-ST-elevation myocardial infarction (NSTEMI) No Yes	47	33 (70.2) 14 (29.8)	16 (64.0) 9 (36.0)	17 (77.3) 5 (22.7)	0.36
Highest troponin level Normal range (0-.04 ng/ml) Not normal range (>.04 ng/ml)	39	16 (41.0) 23 (59.0)	8 (34.8) 15 (65.2)	8 (50.0) 8 (50.0)	0.51
Highest troponin level Less than 0.40 ng/mL 0.40 ng/mL and higher	39	32 (82.0) 7 (18.0)	17 (73.9) 6 (26.1)	15 (93.8) 1 (6.2)	0.21
Venous thromboembolism No Yes	46	40 (87.0) 6 (13.0)	21(87.5) 3(12.5)	19 (86.4) 3 (13.6)	>0.99
Pulmonary embolism No Yes	45	40 (88.9) 5 (11.1)	21 (87.5) 3 (12.5)	19 (90.5) 2 (9.5)	>0.99
Upper GI bleeding No Yes	47	43 (91.5) 4 (8.5)	21 (87.5) 3 (12.5)	22 (96.0) 1 (4.0)	0.61
Lower GI bleeding No Yes	47	42 (89.4) 5 (10.6)	21 (87.5) 3 (12.5)	21 (91.3) 2 (8.7)	>0.99
Hemorrhagic or ischemic stroke No Yes	47	42 (89.4) 5 (10.6)	20 (83.3) 4 (16.7)	22 (95.7) 1 (4.3)	0.35
*Treatments*					
Invasive ventilation No Yes	46	8 (17.4) 38 (82.6)	2 (8.3) 22 (91.7)	6 (27.3) 16 (72.7)	0.13
Non-invasive ventilation No Yes	47	23 (48.9) 24 (51.1)	14 (56.0) 11 (44.0)	9 (40.9) 13 (59.1)	0.38
Self-proning No Yes	47	10 (21.3) 37 (78.7)	5 (20.8) 19 (79.2)	5 (21.7) 18 (78.3)	>0.99
Rotoprone bed No Yes	45	21 (46.7) 24 (53.3)	8 (34.8) 15 (65.2)	13 (59.1) 9 (40.9)	0.14
Steroids No Yes	48	14 (29.2) 34 (70.8)	9 (36.0) 16 (64.0)	5 (21.7) 18 (78.3)	0.35
Anticoagulation Therapeutic No Yes	45	11 (24.4) 34 (75.6)	6 (26.1) 17 (73.9)	5 (22.7) 17 (77.3)	>0.99
Anticoagulant medication Heparin Lovenox NOACs	34	21 (61.8) 9 (26.5) 4 (11.7)	14 (82.4) 3 (26.5) 0 (0.0)	7 (41.2) 6 (35.3) 4 (23.5)	**0.03**
Heparin vs others Heparin Other anticoagulants	34	21 (61.8) 13 (38.2)	14 (82.3) 3 (17.7)	7 (41.2) 10 (58.8)	**0.03**
Primary antiviral medication No Yes	41	31 (75.6) 10 (24.4)	17 (77.3) 5 (22.7)	14 (73.7) 5 (26.3)	>0.99
Tocilizumab No Yes	44	13 (29.5) 31 (70.5)	8 (36.4) 14 (63.6)	5 (22.7) 17 (77.3)	0.51
Length of stay ICU Mean (SD)	45	18.2 (16.7)	16.9 (14.7)	19.7 (18.9)	0.80^a^
Readmissions No Yes	23			16 (69.6) 7 (30.4)	N/A

^a^ Wilcoxon rank-sum test

^b^ Fisher’s exact test

Patients with unavailable information of a variable were excluded from analysis.

N/A = Not applicable

ICU = Intensive Care Unit

**Table 2.** Adjusted and unadjusted odds ratios of the association between ICU mortality and COVID-patient characteristics. N=47 March 2020-May 2020

In-hospital mortality	uOR	95% CI	p-value	aOR	95% CI	p-value
Age	1.0	0.9, 1.1	.077	1.1	1.01, 1.2	**0.03**
Sex Male (ref.) Female	1.0 2.6	0.2, 2.3	.280	1.0 0.2	0.03, 1.1	0.06
Essential hypertension No (ref.) Yes	1.0 4.4	1.1, 17	.033	1.0 6.2	1.1, 34.5	**0.04**
Diabetes Type 2 No (ref.) Yes	1.0 5.4	1.5, 19.3	.009	1.0 5.7	1.2, 29.1	**0.03**

uOR = unadjusted odds ratio; aOR = adjusted odds ratio; CI = Confidence Interval; ref. = reference group.

## DISCUSSION

This cross-sectional study examined the risk factors associated with in-hospital mortality in patients with severe COVID-19 pneumonia, who needed admission to the ICU for higher intensity management from March 1 to May 30, 2020. Specifically, we focused on exploring the associations between patient characteristics, comorbidities, and laboratory abnormalities and in-hospital mortality. The findings indicated a mortality rate of 52%, which is in line with earlier reports reflecting the period before COVID-19 deaths peaked in April 2020. For instance, in a study of patients admitted to a hospital in Detroit (Michigan) between March 1 and April 5, 2020, the 30-day mortality rate for patients in ICU was 40.4%.^16^ Similarly, in a cohort of patients at a large New York medical center, Argenziano and colleagues reported a 43.6% rate of ICU mortality rate between March 1 and April 5, 2020.^22^

After adjusting for age, gender, and diabetes, the study results showed that patients with hypertension had a higher mortality risk than their counterparts. This finding is consistent with previous studies, such as a meta-analysis that found hypertension to be a common underlying condition in all patients across all studies (39.9%), with a higher prevalence (56.8%) in the non-survival groups.^4^ The authors postulated that many infections, including those caused by the severe acute respiratory syndrome (SARS) family of viruses, can result in endothelial dysfunction. As a result, nitric oxide production is reduced and inflammatory markers like CRP, ICAM- 1, and VCAM-1 are released. It is believed that this endothelial dysfunction, caused by decreased nitric oxide bioavailability, is a precursor to both hypertension and diabetes. Endothelial damage produces nitric oxide free radicals, which have a vasodilating effect when they are diminished (in this case due to COVID, an endothelial disease). This will impair its vasodilation effect and render it unable to compete with the endothelial vasoconstrictors, resulting in the vasoconstriction that triggers hypertension and diabetes.^23^ COVID-19 infection can be associated with substantial mortality in patients with diabetes because it worsens preexisting endothelial dysfunction.^4^

Moreover, after adjusting for age, gender, and hypertension, this study found that patients with type 2 diabetes had a higher likelihood of dying than those without diabetes. This is consistent with the findings of a systematic review by Abdi et al. (2020), which also demonstrated that diabetes increased the severity and mortality of COVID-19 patients.^24^ As mentioned above, endothelial dysfunction that is caused by inflammation and reduced nitric oxide in COVID-19 patients increases the risk of thromboembolic complications and damage to vital organs. In patients with significant comorbidities, such as poorly controlled diabetes or hypertension, these effects are aggravated by pre-existing organ damage. Diabetes-related underlying pro-inflammatory states amplify the cytokine storm, which is responsible for multi-organ dysfunction, secondary to ARDS in COVID-19.^24^ Furthermore, diabetes is linked to higher plasminogen levels, which have been postulated to increase the virulence of SARS CoV-2.^25^ Previous studies have proposed that several medications used in patients with COVID-19, such as systemic corticosteroids or antiviral agents, may worsen glycemic control in these patients, which may lead to worse outcomes.^26^

Contrary to the authors’ assumptions, there was no difference in ICU mortality between White and Black/AA patients. This finding is consistent with previous research conducted in Metropolitan Detroit during the first month of the pandemic.^16^ A similar cohort study in the United States with 92 hospitals in 12 states (N =11,210), found no statistically significant difference in in-hospital all-cause mortality between White and Black/AA patients after adjusting for other confounding factors.^27^ However, as previously discussed in the introduction, there are conflicting studies’ findings regarding COVID-19 mortality rate and race disparities.^15,16,18^ The context of the clinical sample including age, the severity of illness, and critically important the time frame of COVID-19 cases are relevant factors when comparing study findings. To our understanding this is the first study of factors associated with mortality among ICU-pneumonia COVID patients during the first wave of COVID in Flint, Michigan.

It is crucial to note that our study’s small sample size may have influenced our power to detect significant differences; our study aimed to identify the characteristics associated with ICU mortality. Further research is needed to thoroughly investigate the factors associated with hospital mortality stratified by race (for example, in severity of the disease such as CCI or the time of admission to the ICU). It was beyond the scope of this study.

Even though we did not find any racial disparities on the first COVID-19 wave, the distribution of patients admitted to the ICU during the study period was 51% Black/AA and 49% White compared to 78% White and 18% Black/AA during the last fiscal year 2022. This change in the racial distribution of ICU patients may show how COVID-19 disproportionately affected the Black/AA community in Flint, but additional research is required to confirm this hypothesis. One of the limitations of this study was that key factors, such as zip code and other proxies of socioeconomic status were not included in the study design; it was out of the scope of this study. Future research may focus on the socioeconomic and other environmental factors that may act as moderators or mediators between new cases of COVID-19 and COVID-19 mortality rate in Flint during various waves.

Results should not be generalized to the level of individual cities, states, or the entire country due to several sample-related limitations for two main reasons. First, during COVID-19 wave-1, this study included only patients from a single community hospital in Flint. In the United States, the term “wave-1” has been defined using various criteria and time frames. We selected March to May because it was the period with highest COVID-19 ICU census. Second, we focused our study on severe COVID-19 pneumonia, a subpopulation with one of the highest mortality rates. The restrictive inclusion criteria led to the small size of the study sample. Nevertheless, by focusing on an underserved community receiving health care at a community-based teaching hospital our research findings could be generalizable to a larger segment of the US population that receive health care at these types of facilities rather than at large academic affiliated-research intensive health care system. Therefore, our findings could provide a closer real-life picture for underserved populations and resource limited health care facilities.

COVID -19 virus continues to mutate into other variants, infecting people and in some cases, resulting in long COVID, hospitalizations, and even death. The CDC state-level forecast has reported an increase of hospitalizations from August 2023 to October 2023 in Michigan (www.cdc.gov/coronavirus). Stating the factors that could increase morbidity and mortality among COVID-19 patients in 2020, it is still relevant especially for health care providers and patients with diabetes and/or hypertension. These high-risk groups need to acknowledge themselves and take all prophylactic measures. COVID 19 is not declared a public health emergency, the federal Public Health Emergency (PHE) for COVID-19 expired in May 2023, which means treatments and testing might become a financial burden on the public and less access to diagnostic testing. Therefore, more unrecognized/undiagnosed cases, and more infected people in the community, and unfortunately more morbidity and mortality; particularly among those who are at elevated risk.

## CONCLUSION

In conclusion, the findings of this cross-sectional study of patients admitted to the ICU of a hospital in Flint with severe COVID-19 pneumonia support the evidence that comorbid conditions such as type 2 diabetes and hypertension contribute to poorer outcomes in these patients. Contrary to the investigators’ hypotheses, no racial differences in ICU mortality rates were found to be statistically significant. Additional large scale, in-depth studies are needed to determine whether the novel COVID-19 virus strains will exhibit comparable trends.

**This project was completed at McLaren Flint, Michigan. The principal author Dr. Atefeh Kalantary is now at the Adventist Health Simi Valley, California; and Dr. Arunima Dutta, is an Internist at the Virginia Mason Franciscan Health, Seattle, Washington.

### Conflict of Interest

None
